# An apple (*Malus domestica*) AP2/ERF transcription factor modulates carotenoid accumulation

**DOI:** 10.1038/s41438-021-00694-w

**Published:** 2021-10-05

**Authors:** Qingyuan Dang, Haiyun Sha, Jiyun Nie, Yongzhang Wang, Yongbing Yuan, Dongjie Jia

**Affiliations:** Qingdao Key Lab of Modern Agriculture Quality and Safety Engineering, College of Horticulture, Qingdao Agricultural University, Qingdao, 266109 China

**Keywords:** Transcriptional regulatory elements, Plant molecular biology

## Abstract

Color is an important trait for horticultural crops. Carotenoids are one of the main pigments for coloration and have important implications for photosynthesis in plants and benefits for human health. Here, we identified an APETALA2 (AP2)/ETHYLENE RESPONSE FACTOR (ERF) transcription factor named MdAP2-34 in apple (*Malus domestica* Borkh.). *MdAP2-34* expression exhibited a close correlation with carotenoid content in ‘Benin Shogun’ and ‘Yanfu 3’ fruit flesh. MdAP2-34 promotes carotenoid accumulation in MdAP2-34-OVX transgenic apple calli and fruits by participating in the carotenoid biosynthesis pathway. The major carotenoid contents of phytoene and β-carotene were much higher in overexpressing MdAP2-34 transgenic calli and fruit skin, yet the predominant compound of lutein showed no obvious difference, indicating that MdAP2-34 regulates phytoene and β-carotene accumulation but not lutein. *MdPSY2-1* (phytoene synthase 2) is a major gene in the carotenoid biosynthesis pathway in apple fruit, and the *MdPSY2-1* gene is directly bound and transcriptionally activated by MdAP2-34. In addition, overexpressing *MdPSY2-1* in apple calli mainly increases phytoene and total carotenoid contents. Our findings will advance and extend our understanding of the complex molecular mechanisms of carotenoid biosynthesis in apple, and this research is valuable for accelerating the apple breeding process.

## Introduction

Color is an important trait for horticultural crops, such as fruits, vegetables, and flowers^1^. Carotenoids are one of the main pigments for coloration, showing yellow, orange, red, or others in horticultural reproductive organs^2^. Carotenoids are important components for photosynthesis and are involved in light harvesting and photoprotection, plant growth, and development^3^. Carotenoids are valuable molecules that are beneficial for human health and animal survival, and carotenoids are also precursors of provitamin A and are antioxidants for reducing various chronic diseases^4,5^.

The biosynthetic pathway of carotenoids is well known in a variety of plant species, and it is modulated by many factors, such as limiting and metabolic enzyme steps, the availability of storage organs and structures, or transcription factors (TFs)^2,6,7^. The structural genes encoding metabolic enzymes in the carotenoid pathway, including *phytoene synthase* (*PSY*), *phytoene desaturase* (*PDS*), *ζ-carotene isomerase* (*ZISO*), *zeta-carotene desaturase* (*ZDS*), *carotenoid isomerase* (*CRTISO*), *lycopene β-cyclase* (*LCY-β*), *lycopene ε-cyclase* (*LCY-ε*), *beta-carotene hydroxylase* (*CHY-β*), *zeaxanthin epoxidase* (*ZEP*), *carotenoid cleavage dioxygenases* (*CCD*), and *9-cis-epoxycarotenoid dioxygenase* (*NCED*), have previously been characterized^2^. Previous studies have shown that PSYs play a significant role in carotenoid accumulation^8,9^. Transgenic tomato fruits and potato tubers overexpressing *PSY* show increased levels of total carotenoids^10,11^. Multiple *PSY* genes modulate carotenoid accumulation in many species, such as maize, rice, loquat, and apple^12–15^. CaPSY2 can increase carotenoid content and compensate for the absence of PSY1 in pepper (*Capsicum* spp.) fruit^16^. OR/OR-like proteins influence carotenoid content by affecting PSY protein levels through posttranscriptional regulation in *Arabidopsis* and sweet potato^17,18^. For the other limiting genes, the downregulation of *LCY-ε* increases the β-carotene content in potato^19^. The expression of the *NCED* gene results in a low β-carotenoid content in the white apricot cultivar^20^. Inhibition of *BoaCRTISO* expression in Chinese kale affected carotenoid pathways and led to decreased carotenoid concentrations^21^. The *Orange* gene *CmOr* regulates β-carotene accumulation in melon fruit by repressing β-carotene turnover and posttranslationally enhancing carotenogenesis in chromoplasts^22^.

A number of TFs have been identified to be involved in the carotenoid pathway. AGAMOUS-like 1 and FRUITFULL, which belong to MADS-box TFs, regulate carotenoid content in tomato^23^, and CsMADS6 modulates carotenoid metabolism by directly regulating *LCYb1* in sweet orange^24^. The NAC TFs SlNAC1, SlNAC4, and CpNAC1 regulate carotenoid accumulation during tomato and papaya fruit ripening^25–27^. MYB TFs, RCP1 in *Mimulus lewisii* flowers^28^, CrMYB68 in citrus^29^, and AdMYB7 in kiwifruit^30^, are all involved in carotenoid regulation. The R2R3-MYB TF WP1 together with MtTT8 and MtWD40-1 promotes floral carotenoid accumulation by directly activating the expression of *MtLYCe* and *MtLYCb* in *Medicago* petals^31^. CpbHLH1/2 regulates carotenoid biosynthesis and is related to carotenoid content during fruit ripening of papaya^32^. *Sl*_H2A. Z plays an essential role in the modulation of carotenoid content in tomato^33^.

The APETALA2 (AP2)/ETHYLENE RESPONSE FACTOR (ERF) superfamily contains four subfamilies according to the number of AP2/ERF domains and sequences, including AP2, CBF/DREB, ERF, and RAV^34^. AP2 subfamily proteins contain single or two AP2/ERF domains that are involved in the regulation of development^35^. AP2/ERF proteins can bind to DNA cis-acting elements, including DRE (CCGAC), GCC box (AGCCGCC), (A/G)CC(G/C)AC, and AA(T)TTCAAA^36^. The TFs of the AP2/ERF superfamily play important roles in growth, development, and stress response processes in higher plants^34–37^. PpERF3 activates ABA biosynthesis by positively regulating PpNCED2/3 transcription in peach^38^. AP2/ERF TFs have been reported to modulate carotenoid accumulation. SIERF6 negatively regulates carotenoid accumulation in tomato^39^. In *Arabidopsis*, ERFs and RAP2.2 promote carotenoid biosynthesis by binding to the *PSY* promoter^40^. However, whether and how AP2/ERF TFs modulate carotenoid metabolism in apple fruit are currently unknown.

Apple (*Malus domestica* Borkh.), which has high nutrient value, is one of the most economical fruit crops worldwide. Fruit flavor and color are major apple traits that determine consumer preferences. Because of differences in carotenoid accumulation, the flesh color varies from white to yellow in different apple genotypes^41^. Previous studies indicated that *MdPSY2* played a dominant role in carotenoid accumulation in apple fruits and was activated by AP2/ERF TFs^15,41^. There was a strong positive correlation between the expression of the TF *AP2D26* and *PSY2* in apple fruit^15^. In this study, we found that the total carotenoid contents of ‘Benin Shogun’ fruits were significantly higher than those of ‘Yanfu 3’ fruits during the developmental period at 150 and 170 days after full bloom (DAFB). We characterized an AP2 TF named MdAP2-34 and found that it induced increases in carotenoid accumulation. MdAP2-34 was able to promote carotenoid levels by enhancing *MdPSY2-1* promoter activity. Moreover, overexpressing *MdPSY2-1* in apple calli mainly increases phytoene and total carotenoid contents. Our findings will advance and extend our understanding of the complex molecular mechanisms of carotenoid biosynthesis in apple.

## Materials and methods

### Plant materials

Apple fruits of ‘Benin Shogun’ and ‘Yanfu 3’ were collected at 120, 150, and 170 days after full bloom (DAFB). ‘Benin Shogun’ and ‘Yanfu 3’ are bud mutations of ‘Red Fuji’. Every treatment included six fruits. Leaves of ‘Benin Shogun’ were collected in late spring and used for DNA extraction. Apple fruits of Granny Smith used for the transient transformation were collected at 150 DAFB. The fruits and leaves were stored at –78 °C until use.

Apple calli were induced from the ‘Orin’ apple flesh and were used for the transformation^42^. The calli were cultured and subcultured on MS medium (Murashige and Skoog basal salt mixture) supplemented with 1.0 mg/L 2,4-dichlorophenoxyacetic acid (2,4-D) and 1.0 mg/L 6-benzylaminopurine (6-BA).

### Subcellular localization

The subcellular localization of MdAP2-34 was conducted as previously described^42^. The open reading frame (ORF) sequence of MdAP2-34 without the stop codon was cloned and inserted into the pRI101:GFP expression vector. The fusion construct 35S:MdAP2-34:GFP was transformed into *N. benthamiana* leaf epidermal cells. Fluorescence was detected by confocal microscopy after 2−3 days of transfection. DAPI (4′,6-Diamidino-2-phenylin-dole; Invitrogen) was used to stain the nucleus.

### Total carotenoids extraction and measurement

The extraction and determination of total carotenoids were performed using a method as described previously^43^. Each sample was extracted with acetone-hexane (1:1, vol/vol). The total carotenoid content of the extracts was determined using a UV-2550 UV−vis spectrophotometer.

### Quantitative measurement of carotenoid components

Carotenoid components of transformed apple fruit skin and apple calli were measured by MetWare Company (http://www.metware.cn/)^33^. Carotenoid compounds were detected using the AB Sciex QTRAP6500 LC–MS/MS platform. Three replicates of each assay were performed. Apple fruit skin and apple calli were freeze-dried and then homogenized and powdered in a mill of 50 mg of dried powder. The dried powder was then extracted with a mixed solution of n-hexane:acetone:ethanol. The extracts were analyzed using an LC-APCI-MS/MS system (UHPLC, ExionLC™AD; https://sciex.com.cn/; MS, Applied Biosystems 6500 Triple Quadrupole, https://sciex.com.cn/). The analytical and detection conditions were as follows: HPLC: column, YMC C30 (3 µm, 2 × 100 mm); solvent system, methanol:acetonitrile (3:1, V/V) (0.01% BHT, 0.1% formic acid):methyl tert-butyl ether (0.01% BHT); gradient program, 100:0 V/V at 0–3 min, 100:0 V/V at 3–6 min, 58:42 V/V at 6–8 min, 20:80 V/V at 8–9 min, 5:95 V/V at 9 min, 100:0 V/V at 9.1–11 min; flow rate, 0.8 mL/min; temperature, 28 °C; injection volume, 2 μL. The α-carotene, antheraxanthin, violaxanthin, neoxanthin, phytofluene, phytoene, β-cryptoxanthin, and rubixanthin standards were purchased from BOC Chemicals. The zeaxanthin standard was purchased from Bide Chemicals. The β-carotene standard was purchased from RHAWN Chemicals. The Lutein standard was purchased from Aladdin Chemicals.

### RNA extraction, qRT–PCR assay, and extraction of genomic DNA

Total RNA was isolated from fruit flesh of ‘Benin Shogun’ and ‘Yanfu 3’, fruit skin of transiently transformed ‘Granny Smith’ and transgenic apple calli (TAC), and qRT–PCR (reverse transcription-quantitative PCR) was carried out as previously described^44^. *EF1*α was used to serve as the reference gene. The qRT–PCR assays were conducted with three biological replicates, and each biological replicate was conducted with three technical replicates. Genomic DNA was isolated form ‘Granny Smith’ leaves. All primers are used in Supplemental Table S1.

### Construction of plasmids and genetic transformation

The overexpression and interference plasmids were constructed as previously described^45^. Each ORF sequence of *MdAP2-34* and *MdPSY2-1* was cloned and inserted into pRI101-FLAG (overexpression vector). A 728 bp fragment (located between +5 and +732 bp of the ORF) of MdAP2-34 was cloned and inserted into pFGC5941 (RNAi vector). Every recombinant plasmid of 35S:MdAP2-34, RNAi:MdAP2-34, and 35 S:MdPSY2-1 was then transformed into Granny Smith apple fruit skin and Orin apple calli^45^. All primers are used in Supplemental Table S1.

### Proteins extraction and western blotting

Protein extraction and western blotting assays were performed as previously described^42,45^. Anti-ACTIN antibody and anti-FLAG antibody were used, which were obtained from Beyotime Biotech Company.

### Yeast one-hybrid assay

Y1H (yeast one-hybrid) assays were conducted as previously described^45^. The ORF sequence of *MdAP2-34* was cloned and inserted into the vector pJG4-5 (the effector vector) (Clontech, USA). Each promoter fragment (upstream of the ATG codon) of *MdPSY1* (1725 bp), *MdPSY2-1* (1733 bp), *MdPSY2-2* (1850 bp), *MdPDS* (1659 bp), *MdZDS1* (1800 bp), *MdZDS2* (1683 bp), *MdLCY-β* (1748 bp), *MdCHY-β2-1* (1631 bp), *MdZEP* (1758 bp), and *MdCCD1* (1782 bp) was cloned into the vector pLacZi (the reporter vector). All primers are used in Supplemental Table S1.

### EMSAs

The EMSA was conducted as previously described^45^. The ORF sequence of *MdAP2-34* was cloned into the vector pGEX-4T-1 (with GST-tag) and then transformed into BL21 (DE3) *Escherichia coli* cells for MdAP2-34 protein expression. The 5ʹ biotin end-labeled probes were synthesized by Sangon Biotech. The EMSA was performed using the LightShift™ Chemiluminescent EMSA Kit (Thermo Fisher Scientific, USA). The primers were used, and the biotin-labeled sequences of the *MdPSY2-1* promoter are shown in Supplemental Table S1.

### ChIP-PCR assay

The ChIP-PCR assay was performed following Jia et al.^45^. The ORF of *MdAP2-34* was inserted into the pRI101-FLAG vector (with 3×FLAG), and the fusion MdAP2-34-FLAG was generated. Recombinant pRI101-FLAG-MdAP2-34 was transformed into apple calli, and the MdAP2-34-FLAG protein was extracted from transgenic calli. ChIP-PCR analysis was conducted using the EpiQuik™ Plant ChIP Kit (Epigentek, USA) and anti-DDDDK tag (Binds to FLAG® tag sequence) antibody (ab125243, Abcam, UK). Three promoter regions of *MdPSY2-1* were used to assess their enrichment. Every ChIP assay was conducted three replicates. The primers used are in Supplemental Table S1.

### LUC assay

The LUC analysis was conducted as previously described^45^. The ORF of *MdAP2-34* was inserted into pGreenII 62-SK (the effector vector) under the control of the CaMV35S promoter. The promoter fragment of *MdPSY2-1* was inserted into pGreenII 0800-LUC (the reporter vector). The vectors of the effector and reporter were cotransformed into 4-week-old *N. benthamiana* leaves. The luciferase signals of Firefly and Renilla were observed and measured using an Infinite M200 (Tecan, Switzerland). Every LUC analysis was conducted six replicates. The primers used are in Supplemental Table S1.

### Statistical analysis

In the experimental design used, the data were tested and analyzed for three biological replicates, and each biological replicate was analyzed three technical repetitions. Significant differences were determined using GraphPad Prism 5 software^42,45^ (* represents *P* < 0.05; ** represents *P* < 0.01; *** represents *P* < 0.001).

## Results

### Characterization of MdAP2-34 associated with carotenoid content in ‘Benin Shogun’ and ‘Yanfu 3’ apple fruits

**Fig. 1:**
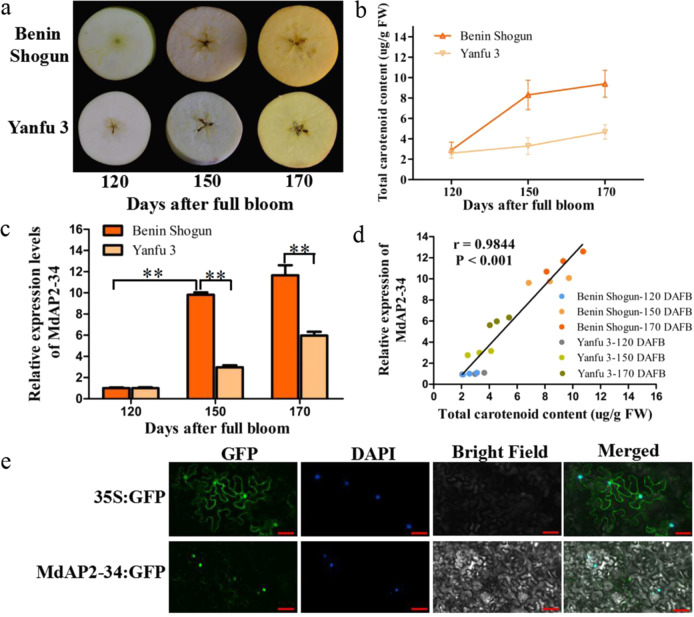
MdAP2-34 was identified as a candidate gene associated with carotenoid content. **a** Phenotypes of ‘Benin Shogun’ and ‘Yanfu 3’ fruits at 120, 150, and 170 DAFB. **b** The total carotenoid content in ‘Benin Shogun’ and ‘Yanfu 3’ fruit flesh at 120, 150, and 170 DAFB. **c** The transcript level of *MdAP2-34* in ‘Benin Shogun’ and ‘Yanfu 3’ fruit flesh at 120, 150, and 170 DAFB. **d** Correlation analysis between the expression of *MdAP2-34* and fruit carotenoid content in ‘Benin Shogun’ and ‘Yanfu 3’ fruit flesh. **e** Subcellular localization of transiently expressed MdAP2-34:GFP fusion protein and the control 35S:GFP in *Nicotiana benthamiana* leaves. 6-Diamidino-2-phenylindole (DAPI) was used to stain the nucleus. Fluorescence images were observed and obtained using confocal microscopy. Scale bars = 50 µm. Values are the mean ± SE in (**b**, **c**)

A previous study indicated that the AP2 subfamily consists of 51 genes in apple, which contain single or double AP2 domain^34^. Based on the annotation of the website (http://bioinformatics.cau.edu.cn/AppleMDO/)^46^, 12 AP2 TFs were identified that could be expressed in apple fruits (Supplemental Table S2). In the ‘Benin Shogun’ and ‘Yanfu 3’ fruit flesh (Fig. 1a), the total carotenoid content increased during the developmental period at 120, 150, and 170 DAFB (Fig. 1b). The total carotenoid content of ‘Benin Shogun’ fruits was significantly higher than that of ‘Yanfu 3’ fruits at 150 and 170 DAFB (Fig. 1b). Pearson’s correlation (*r*) between each AP2 TF transcript levels and total carotenoid content in apple flesh of ‘Benin Shogun’ and ‘Yanfu 3’ indicated that the expression level of *MdAP2-34* was significantly positively associated with carotenoid contents (Supplemental Table S2). However, the *MdAP2-24* expression level was inconsistent with the total carotenoid content in ‘Yanfu 3’ fruit flesh, and the *MdAP2-44* expression level was negatively correlated with the total carotenoid content, so the two genes were filtered out. The transcript level of *MdAP2-34* increased in both ‘Benin Shogun’ and ‘Yanfu 3’ fruit flesh during the developmental period at 120, 150, and 170 DAFB (Fig. 1c). The expression level of *MdAP2-34* in ‘Benin Shogun’ fruits was significantly higher than that in ‘Yanfu 3’ fruits at 150 and 170 DAFB (Fig. 1c). Furthermore, the expression of *MdAP2-34* showed a close correlation (*r* = 0.9844, *P* < 0.001) with carotenoid content in ‘Benin Shogun’ and ‘Yanfu 3’ fruit flesh (Fig. 1d). Therefore, *MdAP2-34* was selected as a candidate gene associated with carotenoid accumulation.

MdAP2-34 (MD17G1226700 or MDP0000231748) is a typical AP2/ERF TF. MdAP2-34 contains an ORF of 1,101 bp and encodes a predicted 367 amino acid (aa) protein. MdAP2-34 was predicted by NCBI (https://www.ncbi.nlm.nih.gov/Structure/cdd) to contain two conserved AP2 domains (49-121 aa, 151-13 aa) (Supplemental Fig. S1a). Phylogenetic analysis showed that MdAP2-34 and AtAIL5 have high homology (Supplemental Fig. S1b), and previous results indicated that AtAIL5 could induce a larger floral organ phenotype^47^. To investigate the subcellular localization of the MdAP2-34 protein, the ORF of *MdAP2-34* was fused to EGFP (MdAP2-34:GFP) and transiently expressed in *Nicotiana benthamiana* leaves. The vector pRI101-GFP (35S:GFP) was used as a control. The results indicated that fusion protein signals were identified in the nucleus (Fig. 1e).

### MdAP2-34 positively regulates carotenoid accumulation in transgenic lines

**Fig. 2:**
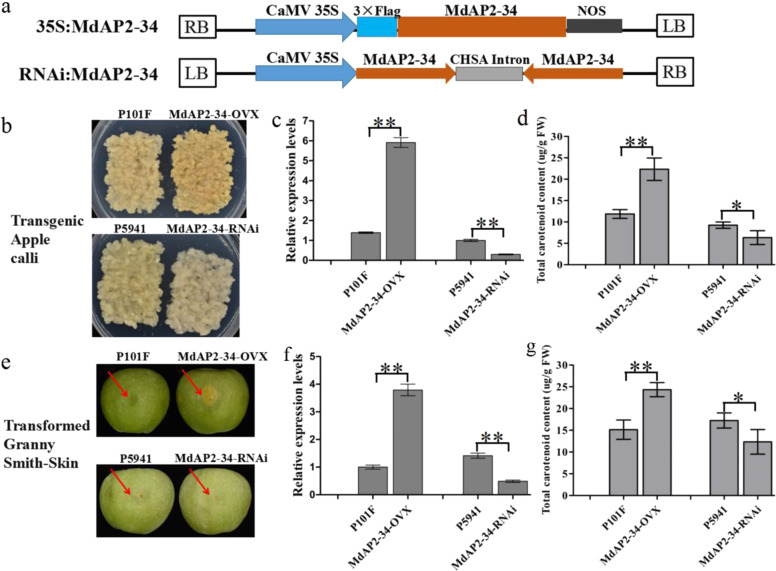
MdAP2-34 positively regulates carotenoid contents in transgenic apple calli and fruit skin. **a** T-DNA region of the expression vectors 35S:MdAP2-34 and RNAi:MdAP2-34 used for transformation. LB, left border; RB, right border; CaMV35S, CaMV (Cauliflcwer mosaic) 35S promoter; NOS, Terminater of synthase. *MdAP2-34* was overexpressed (MdAP2-34-OVX) and silenced (MdAP2-34-RNAi) in apple calli (**b**) and in the fruit skin of Granny Smith (**e**). Transcripts of *MdAP2-34* in transgenic apple calli (**c**) and in Granny Smith fruit skin (**f**). The total carotenoid content in transgenic apple calli (**d**) and in Granny Smith fruit skin (**g**). Values are the mean ± SE in (**c**, **d**, **f**, and **g**)

To confirm whether MdAP2-34 could modulate carotenoid accumulation, the constructs of 35S:MdAP2-34 and RNAi:MdAP2-34 (Fig. 2a) were transformed into apple calli, and the empty vectors of pRI101-flag (P101F) and pFGC5941 (P5941) served as controls (Fig. 2b). The MdAP2-34-FLAG protein was extracted and detected in *MdAP2-34*-overexpressing transgenic calli (Supplemental Fig. S2). The *MdAP2-34* expression level was remarkably higher in MdAP2-34-OVX transgenic calli than in P101F calli but much lower in the MdAP2-34-RNAi calli than in P5941 calli (Fig. 2c). The total carotenoid content was significantly higher in MdAP2-34-OVX calli (22.33 µg/g FW) than in P101F calli (11.87 µg/g FW), with an increase of 88.12% (Fig. 2d; Table 1). The total carotenoid content was lower in MdAP2-34-RNAi calli (6.33 µg/g FW) than in P5941 calli (9.25 µg/g FW), with a reduction of 31.57% (Fig. 2d; Table 1). For the carotenoid compounds, in total, 12 carotenoids were isolated from TAC (Table 1). Among these carotenoids, β-carotene, β-cryptoxanthin, phytoene, and violaxanthin were the major carotenoids present (Table 1). The contents of four specific carotenoids (β-carotene, β-cryptoxanthin, phytoene, and violaxanthin) were remarkably higher in MdAP2-34-OVX calli than in P101F calli and were much lower in MdAP2-34-RNAi calli than in P5941 calli (Table 1). These results suggest that MdAP2-34 enhances the carotenoid contents in apple calli.

**Table 1:** Carotenoid content (μg/g dry weight) in transformed Granny Smith fruit skin and transgenic apple calli

Compounds	Transformed Granny Smith fruit skin				Transgenic apple calli			
	P101F	MdAP2-34-OVX	P5941	MdAP2-34-RNAi	P101F	MdAP2-34-OVX	P5941	MdAP2-34-RNAi
α-Carotene	1.9800 ± 0.1136	0.8600 ± 0.0866*	1.6084 ± 0.3647	1.5738 ± 0.3975	0.4807 ± 0.0451	0.4313 ± 0.0200	0.4481 ± 0.0607	0.3506 ± 0.0946
β-Carotene	34.1367 ± 1.1502	46.8700 ± 1.2275**	40.5991 ± 2.0013	14.6375 ± 1.1551**	3.3433 ± 0.2201	9.2767 ± 0.2312**	2.9135 ± 0.3018	1.2273 ± 0.4120**
β-Cryptoxanthin	1.5300 ± 0.0624	3.4367 ± 0.4605**	1.6784 ± 0.1460	0.8386 ± 0.3776**	4.2033 ± 0.2074	7.2600 ± 0.4551**	5.9393 ± 0.1893	3.7825 ± 0.8142*
β-Cryptoxanthin laurate	0.2093 ± 0.0166	N/A	0.2568 ± 0.0684	0.2742 ± 0.0865	0.1247 ± 0.0078	N/A	N/A	N/A
(E/Z)-Phytoene	28.4500 ± 0.8088	42.1300 ± 1.6358**	37.5290 ± 1.0611	13.1024 ± 2.3065**	93.9833 ± 1.7061	146.4867 ± 5.2974**	84.0869 ± 1.3419	47.1834 ± 3.3485**
Phytofluene	N/A	N/A	N/A	N/A	5.4600 ± 0.3404	N/A	4.8780 ± 0.4633	5.5239 ± 0.7254
Lutein	119.9633 ± 4.2772	125.3300 ± 4.4951	104.0202 ± 5.6031	102.9335 ± 9.4847	0.0870 ± 0.0102	0.1103 ± 0.0152	0.1215 ± 0.0141	0.0677 ± 0.0133
Lutein dilaurate	0.5983 ± 0.0660	1.0167 ± 0.1441	0.6767 ± 0.2317	0.7778 ± 0.4020	N/A	0.1623 ± 0.0090	N/A	N/A
Lutein dipalmitate	0.1420 ± 0.0154	0.3640 ± 0.0496	0.2002 ± 0.0480	0.2621 ± 0.1890	N/A	N/A	N/A	N/A
Lutein laurate	0.2330 ± 0.0486	0.6033 ± 0.0738	0.2167 ± 0.0471	0.1388 ± 0.0893	N/A	N/A	N/A	N/A
Lutein palmitate	0.2480 ± 0.0655	0.6537 ± 0.0526	0.3050 ± 0.1094	0.3203 ± 0.1057	N/A	N/A	N/A	N/A
Lutein oleate	0.2320 ± 0.0560	0.7747 ± 0.0645	0.3271 ± 0.0487	0.1782 ± 0.0845	N/A	N/A	N/A	N/A
Lutein myristate	0.1797 ± 0.0316	0.3380 ± 0.0493	0.1348 ± 0.0307	0.1386 ± 0.0760	N/A	N/A	N/A	N/A
Neoxanthin	6.2567 ± 0.4626	7.3933 ± 0.3972*	5.4702 ± 0.5422	4.2142 ± 0.7710	N/A	N/A	N/A	N/A
Zeaxanthin	3.2867 ± 0.5159	2.9600 ± 0.2623	4.4042 ± 0.4798	4.5288 ± 0.5534	0.6207 ± 0.0431	0.7100 ± 0.0700	0.5550 ± 0.0652	0.6541 ± 0.0378
Rubixanthin laurate	0.1420 ± 0.0346	N/A	0.1207 ± 0.0288	0.1315 ± 0.0440	N/A	N/A	N/A	N/A
Rubixanthin palmitate	N/A	0.3377 ± 0.0218	N/A	N/A	N/A	N/A	N/A	N/A
Violaxanthin	6.9053 ± 0.7320	22.0220 ± 0.9872**	9.7572 ± 0.6859	2.7087 ± 1.1570**	4.7033 ± 0.1514	9.8067 ± 0.1762**	3.4668 ± 0.4014	2.2722 ± 0.2769*
Violaxanthin laurate	0.0078 ± 0.0022	0.0232 ± 0.0034	N/A	N/A	0.1140 ± 0.0092	0.5030 ± 0.0191	0.1620 ± 0.0144	0.1569 ± 0.0167
Antheraxanthin	0.8290 ± 0.1215	1.6233 ± 0.0833**	1.0031 ± 0.0814	0.9253 ± 0.1469	0.2730 ± 0.0288	0.3193 ± 0.0208	0.3200 ± 0.0355	0.2797 ± 0.0212
Total carotenoids	205.3298 ± 8.5807	256.7366 ± 10.0946**	208.3077 ± 11.5783	147.6841 ± 17.4266**	113.3933 ± 2.7696	175.0663 ± 6.3140**	102.8910 ± 2.8876	61.4983 ± 5.7605**

N/A represents the type of carotenoid was not detected. A significant difference of * represents *P* < 0.05, and ** represents *P* < 0.01

A transient expression assay based on *Agrobacterium tumefaciens* infiltration was used to overexpress or silence *MdAP2-34* in the fruit skin of Granny Smith (Fig. 2e). After 14 days of the transient transformation of Granny Smith, the MdAP2-34-OVX Granny Smith fruit skin was harvested (Fig. 2e). The expression level of *MdAP2-34* was remarkably higher in MdAP2-34-OVX fruit skin than in P101F fruit skin but much lower in MdAP2-34-RNAi fruit skin than in P5941 fruit skin (Fig. 2f). The total carotenoid content was significantly higher in MdAP2-34-OVX fruit skin (24.33 µg/g FW) than in P101F fruit skin (15.15 µg/g FW), with an increase of 60.59% (Fig. 2g; Table 1). The total carotenoid content was lower in MdAP2-34-RNAi fruit skin (12.33 µg/g FW) than in P5941 fruit skin (17.25 µg/g FW), with a reduction of 28.52% (Fig. 2g; Table 1). For the carotenoid compounds, in total, 19 carotenoids were isolated from transgenic Granny Smith fruit skin (Table 1). Among these carotenoids, β-carotene, phytoene, and lutein were the major carotenoids present. The ratios of β-carotene, phytoene, and lutein to total carotenoids in P101F ‘Granny Smith’ fruit skin were 16.63, 13.86, and 58.42%, respectively (Table 1). The contents of four specific carotenoids (β-carotene, β-cryptoxanthin, phytoene, and violaxanthin) were remarkably higher in MdAP2-34-OVX fruit skin than in P101F fruit skin and were remarkably lower in MdAP2-34-RNAi calli than in P5941 calli (Table 1). These findings suggest that MdAP2-34 plays an important role in the carotenoid accumulation and positively regulates carotenoid accumulation in the fruit skin of apple trees.

### Expression of carotenoid biosynthesis genes in transgenic fruit skin and calli

**Fig. 3:**
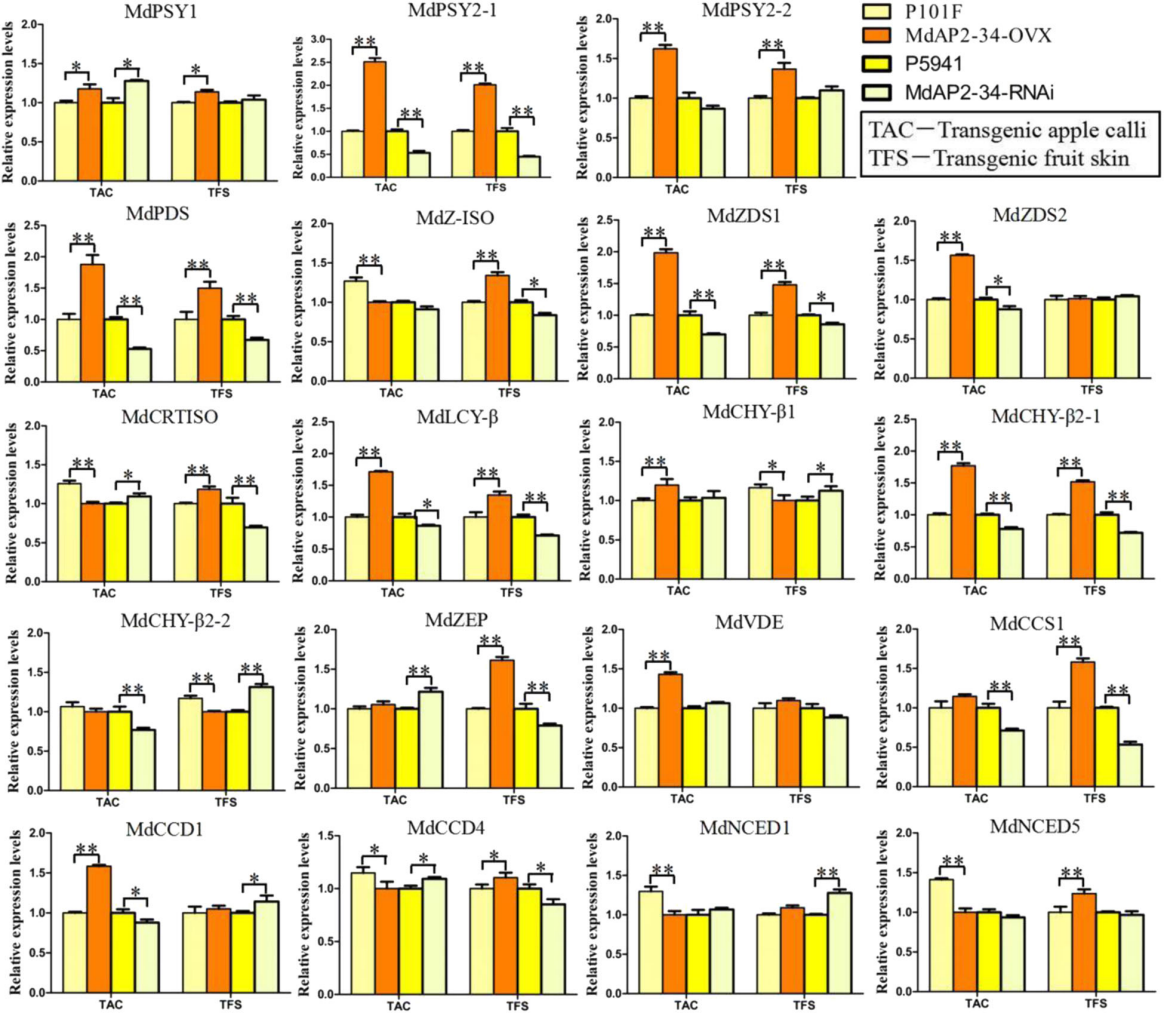
Expression analysis of carotenoid biosynthetic genes in transgenic apple calli and fruit skin. Expression analysis of *MdPAL1*, *MdPSY2-1*, *MdPSY2-2*, *MdPDS*, *MdZDS1*, *MdZ-ISO*, *MdZDS2*, *MdCRTISO*, *MdLCY-β*, *MdCHY-β1*, *MdCHY-β2-1*, *MdCHY-β2-2*, *MdZEP*, *MdVDE*, *MdCCS1*, *MdCCD1*, *MdCCD4*, *MdNCED1*, and *MdNCED5* in transgenic apple calli (TAC) and transgenic ‘Granny Smith’ fruit skin (TFS) using qRT–PCR. Values are the mean ± SE

The structural genes encoding enzymes of carotenoid biosynthesis include *PSY*, *PDS*, *ZISO*, *ZDS*, *CRTISO*, *LCY-β*, *CHY-β*, *ZEP*, *VDE*, *CCS*, *CCD*, and *NCED* (Supplemental Fig. S3), which have previously been characterized^2^. The expression levels of genes related to carotenoid biosynthesis were measured and analyzed in TAC and transgenic Granny Smith fruit skin (TFS) using qRT–PCR (Fig. 3). The expression levels of *MdPSY1*, *MdPSY2-1*, *MdPDS*, *MdZDS1*, *MdZDS2*, *MdLCY-β*, *MdCHY-β2-1*, and *MdCCD1* were significantly higher in MdAP2-34-OVX TAC than in P101F TAC and were remarkably lower in MdAP2-34-RNAi TAC than in P5941 TAC. The expression levels of *MdPSY2-1*, *MdPDS*, *MdZ-ISO*, *MdZDS1*, *MdCRTISO*, *MdLCY-β*, *MdCHY-β2-1*, *MdZEP*, *MdCCS1*, and *MdCCD4* were significantly higher in the MdAP2-34-OVX TFS than in the P101F TFS and were remarkably lower in the MdAP2-34-RNAi TFS than in the P5941 TFS. The expression levels of *MdPSY2-1*, *MdPDS*, *MdZDS1*, *MdLCY-β*, and *MdCHY-β2-1* were markedly upregulated in both TAC and TFS of MdAP2-34-OVX compared to P101F, which were markedly downregulated in both TAC and TFS of MdAP2-34-RNAi compared to P5941 (Fig. 3). These results correspond to the detected differences in carotenoid levels between MdAP2-34-OVX and P101F, and between MdAP2-34-RNAi and P5941 in TAC and TFS, indicating that carotenoid levels are influenced by most carotenoid biosynthetic genes in the carotenoid biosynthesis pathway and a positive correlation between *MdAP2-34* expression levels and those of the genes *MdPSY2-1*, *MdPDS*, *MdZDS1*, *MdLCY-β*, and *MdCHY-β2-1*.

### MdAP2-34 enhances the transcription of *MdPSY2-1* by directly binding to its promoter

**Fig. 4:**
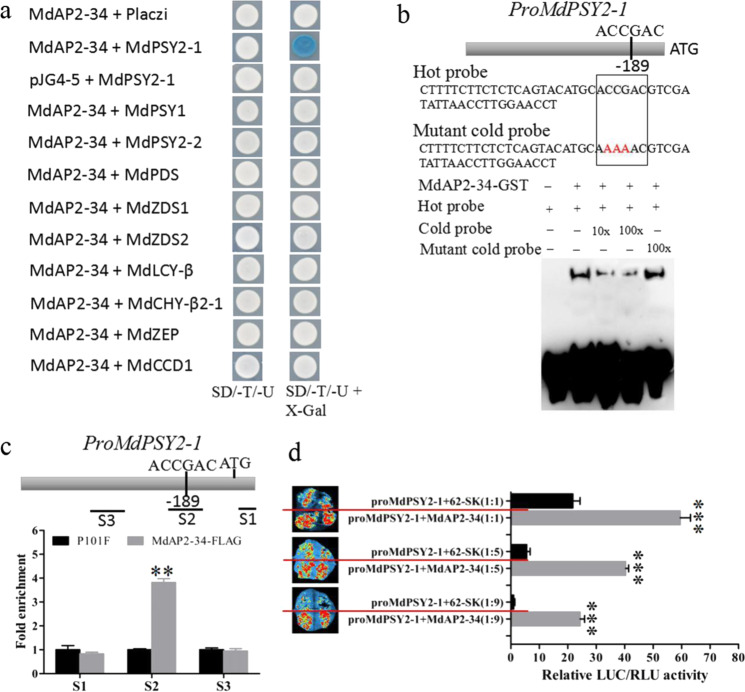
MdAP2-34 enhances the MdPSY2-1 transcription. **a** Y1H assay showing that MdAP2-34 binds to the *MdPSY2-1* promoter. **b** EMSA showing that MdAP2-34 binds to the ACCGAC motif of the *MdPSY2-1* promoter. **c** ChIP-PCR assay showing that MdAP2-34 binds to the *MdPSY2-1* promoter in vivo. Three regions (S1–S3) in *MdPSY2-1* were explored. **d** LUC activity analysis showing that MdAP2-34 enhances the *MdPSY2-1* promoter. Three concentrations of the combination of ProMdPSY2-1:LUC (ProMdPSY2-1) and Pro35S:MdAP2-34 (MdAP2-34) were set up, for which the ratio of ProMdPSY2-1 to MdAP2-34 was 1:1, 1:5, and 1:9. Values are the mean ± SE in (**c,** **d**)

To test how MdAP2-34 modulates carotenoid accumulation, a yeast one-hybrid (Y1H) analysis was conducted to detect whether MdAP2-34 directly regulates the transcription of *MdPSY1*, *MdPSY2-1*, *MdPSY2-2*, *MdPDS*, *MdZDS1*, *MdZDS2*, *MdLCY-β*, *MdCHY-β2-1*, *MdZEP*, and *MdCCD1*. We found that MdAP2-34 was able to directly bind to the *MdPSY2-1* promoter (Fig. 4a). An EMSA was conducted to further confirm the binding interaction. Previous studies reported that many DREB proteins could specifically bind to a core sequence (A/G)CCGAC of the dehydration-responsive element (DRE), such as AtDREB1A and AtDREB2A^48,49^. Here, we found an ACCGAC motif in the *MdPSY2-1* promoter (Fig. 4b). MdAP2-34 was able to bind to the ACCGAC motif in the *MdPSY2-1* promoter (Fig. 4b).

To confirm the binding of MdAP2-34 to the *MdPSY2-1* promoter in vivo, a ChIP-PCR (chromatin immunoprecipitation-PCR) assay was performed. The MdAP2-34-FLAG fusion protein was overexpressed and synthesized in TAC, and P101F was used as a control. The presence of MdAP2-34 enhanced the PCR-based detection of the *MdPSY2-1* promoter (Fig. 4c), indicating that MdAP2-34 can also bind to the *MdPSY2-1* promoter in vivo. Furthermore, to confirm how MdAP2-34 may affect the activity of the *MdPSY2-1* promoter, a luciferase (LUC) transactivation assay in leaves of wild tobacco (*Nicotiana benthamiana*) through cotransformation with Pro35S:MdAP2-34 and ProMdPSY2-1:LUC constructs was conducted. Three combinations of ProMdPSY2-1:LUC (ProMdPSY2-1) and Pro35S:MdAP2-34 (MdAP2-34) were used, including ProMdPSY2-1 to MdAP2-34 ratios of 1:1, 1:5, and 1:9, respectively. When MdAP2-34 was cotransformed with ProMdPSY2-1, *MdPSY2-1* promoter activity was significantly increased. As the ProMdPSY2-1 to MdAP2-34 ratio increased from 1:1 to 1:9, the promoter activity of *MdPSY2-1* was significantly increased (Fig. 4d). Taken together, these results support MdAP2-34 activation of *MdPSY2-1* transcription.

### Overexpressing *MdPSY2-1* in apple calli increases carotenoid content

**Fig. 5:**
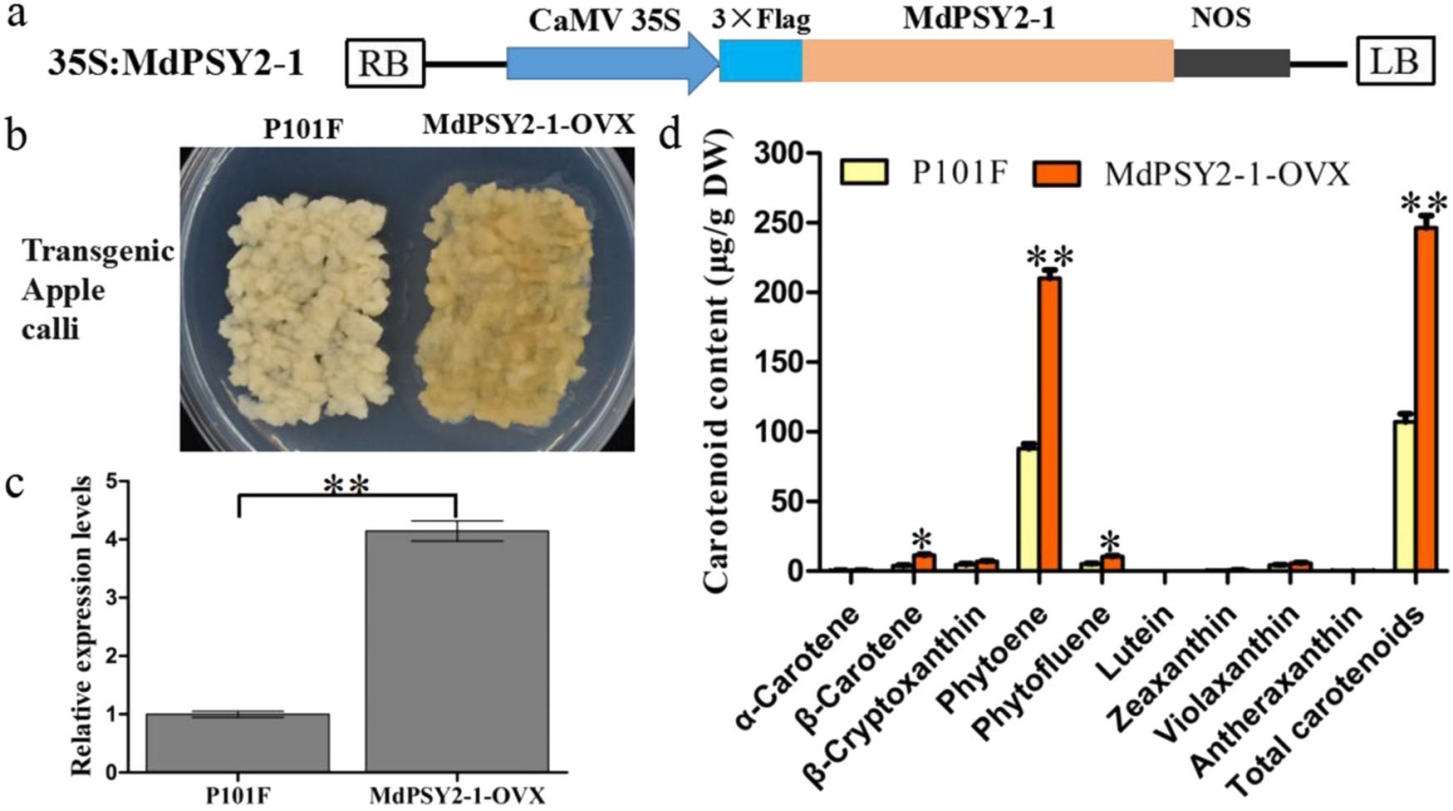
Overexpressing MdPSY2-1 in apple calli. **a** T-DNA region of the expression vector 35S:MdPSY2-1 used for transformation. LB, left border; RB, right border; CaMV35S, Cauliflcwer mosaic 35S promoter; NOS, Terminater of synthase. **b** *MdPSY2-1* was overexpressed (MdPSY2-1-OVX) in apple calli. **c** Transcripts of *MdPSY2-1* in transgenic apple calli. **d** The contents of carotenoid components in transgenic apple calli. Values are the mean ± SE in (**c**, **d**)

The 35S:MdPSY2-1 construct (Fig. 5a) was transformed into apple calli, and the empty vector P101F was used as a control (Fig. 5b). The *MdPSY2-1* expression level was significantly higher in overexpressing transgenic calli (MdPSY2-1-OVX) than in P101F calli (Fig. 5c). The total carotenoid, phytoene, phytofluene, and β-carotene contents were markedly higher in MdPSY2-1-OVX calli than in P101F calli (Fig. 5d). These results indicate that overexpressing *MdPSY2-1* in apple calli mainly increases phytoene, phytofluene, β-carotene, and total carotenoid contents and that *MdPSY2-1* plays an important role in carotenoid accumulation.

## Discussion

**Fig. 6:**
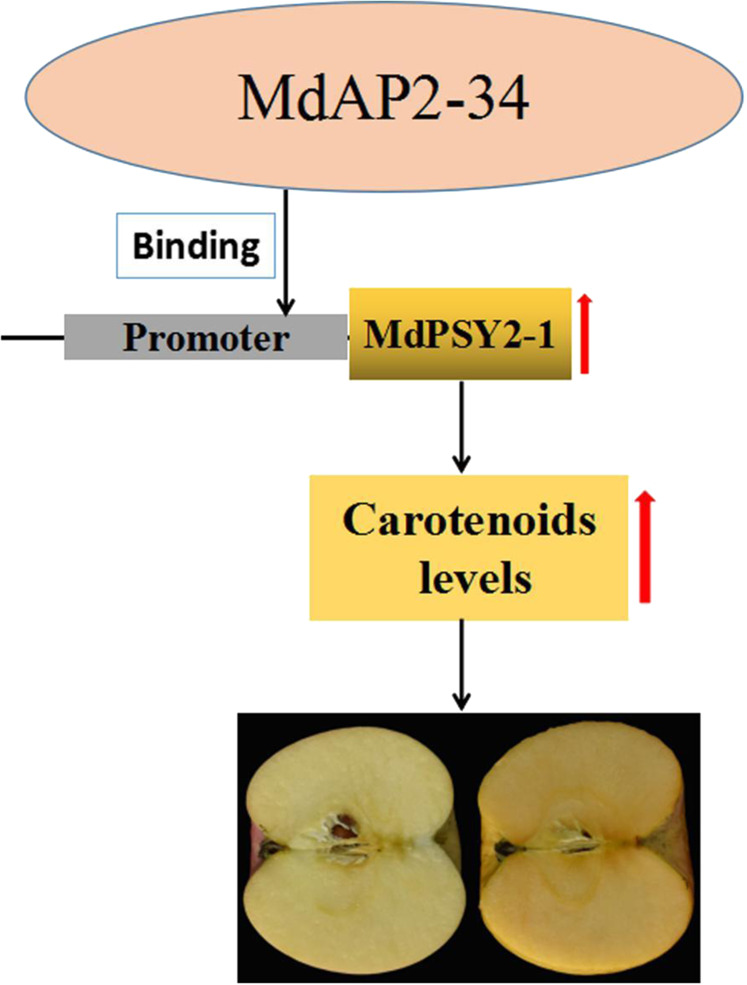
Proposed model for the role of MdAP2-34 in regulating carotenoid accumulation in apple. MdAP2-34 modulates carotenoid accumulation in apple by participating in the carotenoid biosynthesis pathway. MdAP2-34 could modulate carotenoid accumulation by directly binding to the *MdPSY2-1* promoter and enhancing *MdPSY2-1* transcriptional activity

Carotenoids are important pigments for coloration and nutrition in horticultural plants and play important roles in plant growth and development. Apples with healthful metabolites and high nutritional value are consumed worldwide. Recently, carotenoid content and components have become important breeding objectives in apple^30,41^. In this study, we characterized the AP2-type TF MdAP2-34 in Benin Shogun apple, in which ripened fruits appeared orange. Through experiments, we developed a model that explains the molecular basis for MdAP2-34. We found that MdAP2-34 enhances carotenoid accumulation. MdAP2-34 was able to promote carotenoid levels by activating *MdPSY2-1* promoter activity (Fig. 6).

Apples are consumed in large quantities worldwide because of their healthy metabolites. Carotenoid pigments contribute to the skin coloration of apple fruit; however, their concentrations are very low in the apple flesh. A previous study suggested that they have low concentrations of carotenoids in the fruit of commercial apple cultivars (<2.5 µg/g fresh weight) but high fruit carotenoid concentrations in the rootstock cultivar ‘Aotea’^41^. The study indicated that the concentration of total carotenoids in the fruit of the commercial cultivar ‘Benin Shogun’ (with flesh color of orange, Fig. 1a) was up to 9.4 µg/g fresh weight at 170 DAFB, which was much higher than that of ‘Yanfu 3’ and the carotenoid concentrations reported previously. The results suggest that the commercial cultivar ‘Benin Shogun’ may be better for research on the carotenoid metabolic mechanism. Our future study is intended to focus on the mechanisms of carotenoid biosynthesis in different apple cultivars, including the full transcriptome, genetics, and epigenetics. The cause of different genetic variations in carotenoid accumulation in *Malus* will help us to understand the mechanisms of carotenoid accumulation in apple and will be beneficial for the breeding of new apple cultivars with high carotenoid concentrations.

Many TFs play important roles in carotenoid biosynthesis. It is well known that AP2/ERFs participate in many plant development processes and play important roles in regulating fruit quality and pigment biosynthesis^37,50^. However, AP2/ERF TFs have rarely been reported to regulate carotenoid biosynthesis in apple fruits. In this study, based on the previous analysis of the AP2/ERF family^34^ and the annotation of websites (http://bioinformatics.cau.edu.cn/AppleMDO/)^46^, we identified 12 AP2 TFs from the AP2 subfamily expressed in apple fruits. By correlation analysis, we characterized MdAP2-34 as a candidate AP2 TF associated with carotenoid accumulation (Supplemental Table S2). The study only identified the AP2 subfamily, and other AP2/ERF subfamilies may also modulate carotenoid accumulation in apple fruit and requires further research. MdAP2-34 contains two conserved AP2 domains (Fig. 1e); however, the other reported carotenoid-related AP2/ERF TFs contain only one AP2 domain (Supplementary Fig. S1a). MdAP2-34 is a member of the AP2 subfamily, which has high homology with AtAIL5 in *Arabidopsis*, and AtAIL5 could induce a larger floral organ phenotype^47^. MdAP2-34 may have a similar function, and further studies are required to test its function.

It has been reported that AP2/ERF superfamily TFs can modulate carotenoid biosynthesis. With regard to carotenoid regulation, RAP2.2 promotes carotenoid accumulation by binding to the *PSY* promoter in *Arabidopsis*^40^. However, SIERF6 represses carotenoid accumulation in tomato, and reduced *SlERF6* expression results in increased carotenoid concentration^39^. This study indicated that overexpressing *MdAP2-34* in transgenic calli and fruits enhanced carotenoid accumulation (Fig. 2 and Table 1). The contents of four specific carotenoids (β-carotene, β-cryptoxanthin, phytoene, and violaxanthin) were significantly higher in MdAP2-34-OVX fruit skin of transgenic Granny Smith than in P101F fruit skin and were remarkably lower in MdAP2-34-RNAi calli than in P5941 calli (Table 1). The predominant carotenoid compounds differed among different apple cultivars^41^. In ripening apple fruit flesh, the predominant compounds in ‘Royal Gala’ were violaxanthin and neoxanthin, those in ‘Aotea’ flesh were β-cryptoxanthin and β-carotene, and those in ‘Granny Smith’ were lutein. The study also indicated that lutein was the predominant compound in Granny Smith fruit skin, followed closely by β-carotene and phytoene (Table 1). The β-carotene and phytoene contents were much higher in *MdAP2-34-overexpressing* transgenic calli and fruit skin, yet lutein showed no obvious difference, indicating that MdAP2-34 regulates β-carotene and phytoene accumulation but not lutein. MdAP2-34 could not modulate lutein-related genes, and other regulators or proteins may contribute to lutein accumulation in apple, which needs further study.

The structural genes encoding enzymes of carotenoid biosynthesis included *MdPSY2-1*, *MdPDS*, *MdZDS1*, *MdLCY-β*, and *MdCHY-β2-1*, which were markedly upregulated in both MdAP2-34-OVX TAC and fruits and were markedly downregulated in both MdAP2-34-RNAi TAC and fruits (Fig. 3). MdAP2-34 could enhance carotenoid accumulation by activating *MdPSY2-1* promoter activity. However, MdAP2-34 only directly binds to the *MdPSY2-1* promoter but not the other carotenoid-related structural genes (Fig. 4a). There may be other mechanisms by which MdAP2-34 interacts with other TFs or genes to regulate carotenoid biosynthesis. Taken together, carotenoid accumulation in apple fruit is probably controlled by a complex regulatory network and warrants further study.

The genes encoding enzymes of carotenoid biosynthesis include *PSY*, *PDS*, *ZISO*, *ZDS*, *CRTISO*, *LCY-β*, *CHY-β*, *ZEP*, *VDE*, *CCS*, *CCD*, and *NCED*, which have previously been characterized^2^. Multiple *PSY* genes deserve functional diversity, some of which play important roles in carotenoid accumulation^8,9^. Previous studies indicated that PSYs modulate carotenoid accumulation in many fruits of plant species, such as tomato^8^, potato^11^, loquat^14^, apple^15^, and peppers^16^. *MdPSY2* exhibited higher transcript levels than *MdPSY1* in diverse apple cultivars, indicating that *MdPSY2* could be mainly responsible for the first carotenoid pathway step and plays a dominant role in carotenoid accumulation in apple fruits^15,41^. The different expression levels of *MdPSY1* and *MdPSY2* in apple were influenced by their promoters and activated by AP2/ERF TFs. There was a strong positive correlation between the expression of the TF *AP2D26* and *PSY2* in apple fruit, revealing a potential regulatory relationship^15^. The role of PSYs in the carotenoid pathway was prominent in *Arabidopsis*, and the PSY promoter was implicated and activated by AtRAP2.2^40^. The results indicated that *MdPSY2-1* increases carotenoid content and was directly activated by MdAP2-34 (Figs. 4, 5). In addition, posttranscriptional regulation of PSY in controlling carotenoid biosynthesis has been reported, and PSYs are verified as the rate-limiting step in carotenogenesis^17^. In this study, by overexpressing the *MdPSY2-1* gene in apple calli, the total carotenoid and phytoene contents were markedly increased (Fig. 5). The results show that *MdPSY2-1* is a major gene in the carotenoid biosynthesis pathway in apple fruit.

Herein, we found that MdAP2-34 promotes carotenoid accumulation by participating in the carotenoid biosynthesis pathway. *MdPSY2-1* is a major gene in the carotenoid biosynthesis pathway in apple fruit, and the *MdPSY2-1* gene is directly bound and transcriptionally activated by MdAP2-34. Furthermore, overexpressing *MdPSY2-1* in apple calli mainly increases phytoene and total carotenoid contents. This research is valuable for accelerating the apple breeding process and for further understanding the complex mechanisms of carotenoid synthesis in horticultural plants.

## Supplementary information

Supplementary Figures 1-3, Tables 1-2

## Data Availability

All relevant data can be found within the paper and its supporting materials.
