# Supplementary material for: An apple (Malus domestica) AP2/ERF transcription factor modulates carotenoid accumulation
Source: Hortic Res. 2021 Oct 5;8:223. doi: 10.1038/s41438-021-00694-w (PMC8492665; doi:10.1038/s41438-021-00694-w)
Supplement: Supplementary file 1 — Supplementary Figures 1-3, Tables 1-2 [file 41438_2021_694_MOESM1_ESM.pdf]

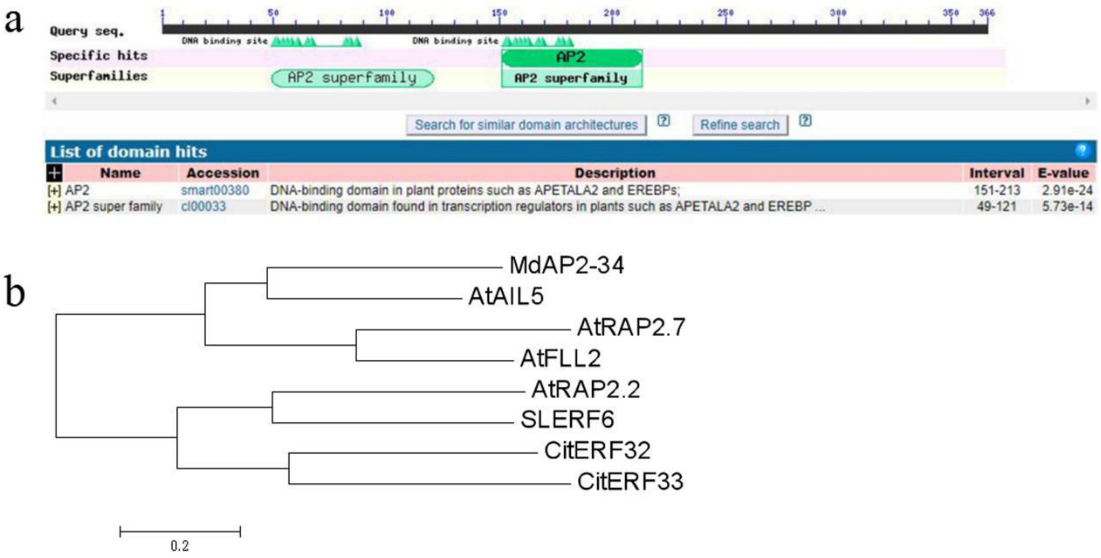

Supplemental Figure S1. Characterization of MdAP2-34. a MdAP2-34 was predicted by NCBI (https://www.ncbi.nlm.nih.gov/Structure/cdd) to contain the two conserved AP2 domains. **b** Phylogenetic analysis was conducted using the protein sequences of MdAP2-34 (MD17G1226700 for *Malus domestica*), AtAIL5 (AT5g57390 for *Arabidopsis thaliana*), AtRAP2.7 (AT2G28550 for *Arabidopsis thaliana*), AtFLL2 (AT4G36920 for *Arabidopsis thaliana*), AtRAP2.2 (AT3G14230 for *Arabidopsis thaliana*), SlERF6 (JN616265.1 for *Solanum lycopersicum*), CitERF32 (Ciclev10005549m.g for *Citrus reticulata*), and CitERF33 (Ciclev10002280m.g for *Citrus reticulata*).

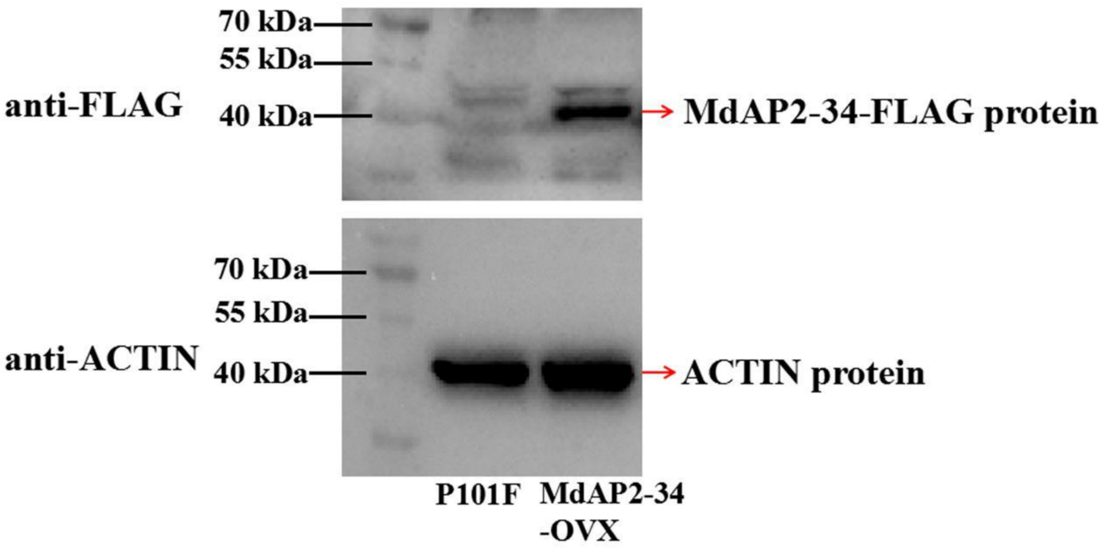

Supplemental Figure S2. Western blotting assay of MdAP2-34-FLAG protein in the overexpressing MdAP2-34 transgenic calli, and P101F calli. The FLAG antibody was used to detect the MdAP2-34-FLAG protein of the transgenic calli. The ACTIN protein was as control. P101F, transformed apple calli with the vector pRI101-FLAG. MdAP2-34-OVX, overexpressing MdAP2-34-FLAG transgenic calli.

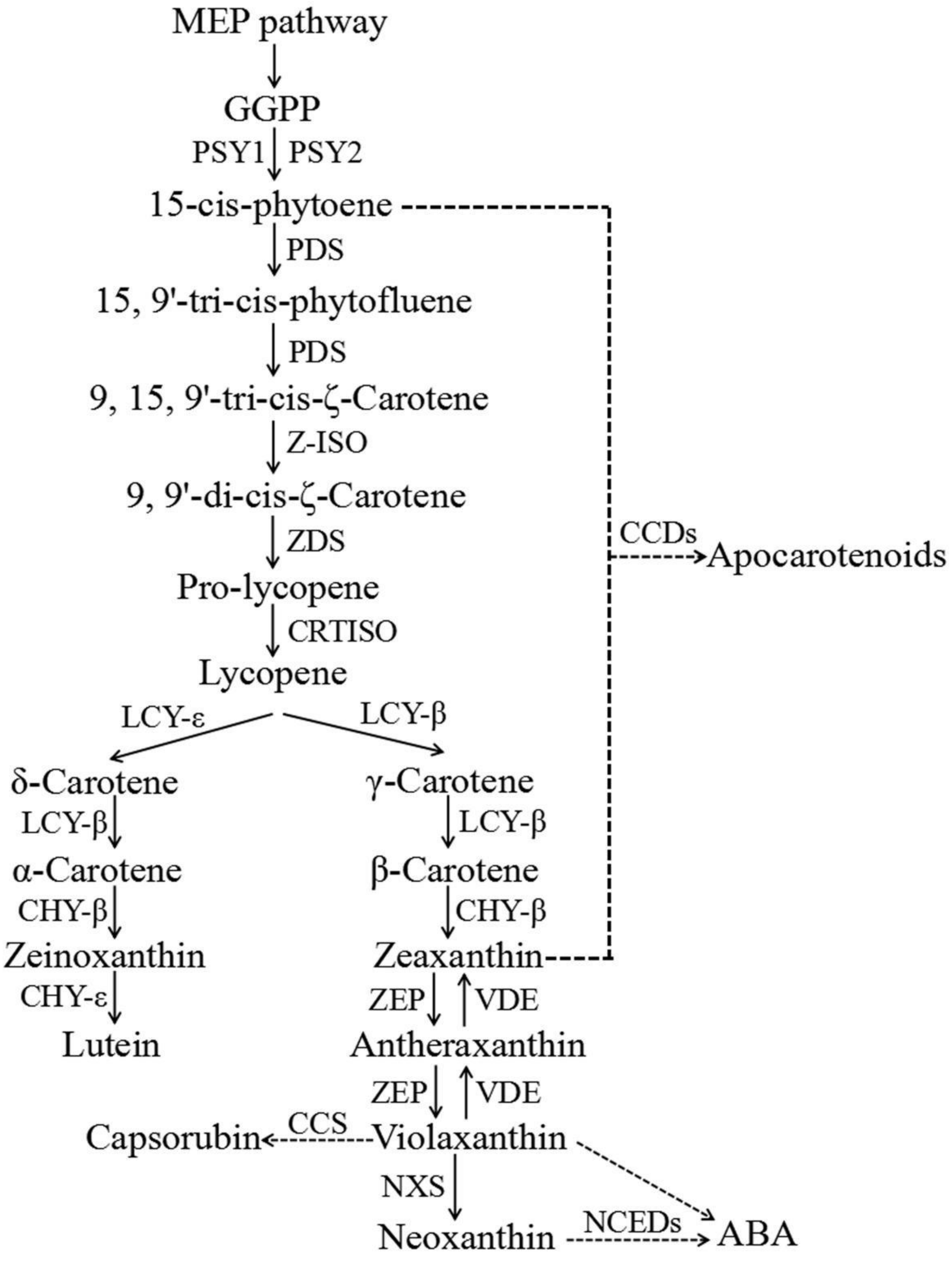

Supplemental Figure S3. Carotenoid biosynthesis pathway. Gene names are indicated for each step of carotenoid biosynthesis pathway. GGPP, geranylgeranyl diphosphate; *PSY*, phytoene synthesis; *PDS*, phytoene desaturase; *ZISO*, ζ-carotene isomerase; *ZDS*, ζ-carotene desaturase; *CRTISO*, carotene isomerase; *LCY-β*, chloroplast-specific lycopene β-cyclase; *LCY-ε*, lycopene ε-cyclase; *CHY-β*, beta-carotene hydroxylase; *CHY-ε*, ε-carotene hydroxylase; *ZEP*, zeaxanthin epoxidase; *VDE*, violaxanthin de-epoxidase; *NXS*, neoxanthin synthesis; *CCS*, capsanthin-capsorubin synthase; *CCD*, carotenoid cleavage dioxygenases; *NCEDs*, 9-cis-epoxycarotenoid dioxygenase.

Supplemental Table S1. The primers used in the study.

Name	Primer sequences (5'->3')	Notes
MdAP2-34_F (ORF, pRI101-FLAG, pRI101-GFP)	ACGCGTCGACATGGCGAAAACCTCACAGC	ORF primers
MdAP2-34_R (ORF, pRI101-FLAG, pRI101-GFP)	GGGGTACCTGCATCAAGTTCACAGTGAAAAAC	ORF primers
MdPSY2-1_F (ORF, pRI101-FLAG)	ACGCGTCGACATGTCAGGTGTTCTACTCTGG	ORF primers
MdPSY2-_R (ORF, pRI101-FLAG)	GGGGTACCTCTAAGCACCAACTGCTTAGTT	ORF primers
MdAP2-34_F1 (RNAi, pFGC5941)	TTGGCGCGCCCGAAAACCTCACAGCAAAACCAG	RNAi constructs
MdAP2-34_R1 (RNAi, pFGC5941)	CATGCCATGGGCCATCAGGGTTTTGCATTACAT	RNAi constructs
MdAP2-34_F2 (RNAi, pFGC5941)	GCTCTAGACGAAAACCTCACAGCAAAACCAG	RNAi constructs
MdAP2-34_R2 (RNAi, pFGC5941)	CCTTAATTAAGCCATCAGGGTTTTGCATTACAT	RNAi constructs
MdAP2-34_F	AGGAAGACAAGGGGCATATGAT	qRT-PCR primers
MdAP2-34_R	ATGGTGTCTTGCTACGCCTC	qRT-PCR primers
MD02G1176000_F (MdAP2-07)	CTCGATGCAACTTGATGCCG	qRT-PCR primers
MD02G1176000_R (MdAP2-07)	AAGCTGCATGGCTTCACTCT	qRT-PCR primers
MD02G1265300_F (MdAP2-23, MdAP2-50)	AGTGAACCAATTGCAACCGC	qRT-PCR primers
MD02G1265300_R (MdAP2-23, MdAP2-50)	ATGGTGCTCCTTCGCATGAC	qRT-PCR primers
MD03G1044200_F (MdAP2-10)	TGTTGCGCACCTACATCACC	qRT-PCR primers
MD03G1044200_R (MdAP2-10)	GTCGTCCTAGGGCACTTGAT	qRT-PCR primers
MD03G1107900_F (MdAP2-41)	TGATCTCCTCCGTCCGGTTA	qRT-PCR primers
MD03G1107900_R (MdAP2-41)	ATGATGCGACGTCGTTTTGC	qRT-PCR primers
MD04G1105200_F (MdAP2-24)	CGACGACGTTTCCATGTCCC	qRT-PCR primers
MD04G1105200_R (MdAP2-24)	GTGCGAGTGGAAAAAGCTGG	qRT-PCR primers
MD07G1099500_F (MdAP2-33)	TTGGACATCCGAAAGAGGGC	qRT-PCR primers
MD07G1099500_R (MdAP2-33)	TTCCGACACCCATTTGGTCC	qRT-PCR primers
MD08G1176000_F (MdAP2-22)	GGATTGCGCGATGTTAAGGC	qRT-PCR primers
MD08G1176000_R (MdAP2-22)	AAGGTTTTGCGCGTGTCTTC	qRT-PCR primers
MD12G1075200_F (MdAP2-44, MdAP2-45)	AGCTTACATTATGGTACAGGCGT	qRT-PCR primers
MD12G1075200_R (MdAP2-44, MdAP2-45)	AAACGGGGCATCTGGTATGG	qRT-PCR primers
MD12G1079800_F (MdAP2-13, MdAP2-30)	TATTGGGGTCCTGGCACTCT	qRT-PCR primers
MD12G1079800_R (MdAP2-13, MdAP2-30)	CAGTTTGGGATGGTTGACGC	qRT-PCR primers
MD12G1125900_F (MdAP2-11)	ATCCGCTGATCACTACGACG	qRT-PCR primers
MD12G1125900_R (MdAP2-11)	GCAATCCATTGCTTCGCAGG	qRT-PCR primers
MD15G1286400_F (MdAP2-17, MdAP2-26)	ATCCAGTTCAAGCTCGTCGG	qRT-PCR primers
MD15G1286400_R (MdAP2-17, MdAP2-26)	GGAGGAGTCTTGCTCCACCG	qRT-PCR primers
MdPSY1_F	AGTGAGAGAACCGAACACGG	qRT-PCR primers
MdPSY1_R	GATTGCCCAAACAGCTCGTC	qRT-PCR primers
MdPSY2-1_F	GGTGAAAGAACAGAGCACGG	qRT-PCR primers
MdPSY2-1_R	TCTGTCCTTCTGCACCACAC	qRT-PCR primers
MdPSY2-2_F	AGGTGTATGACGTGGTGCTG	qRT-PCR primers
MdPSY2-2_R	ATGGCCCTTCTTCTTTCGGG	qRT-PCR primers
MdPDS_F	CAATGCCAAACAAGCCAGGG	qRT-PCR primers
MdPDS_R	ACATAAGCCTGCCCACCAAG	qRT-PCR primers
MdZ-ISO_F	TTCCGTGTTTTGTTTGCGGG	qRT-PCR primers
MdZ-ISO_R	TTGTCAACGGCTGCTACCTC	qRT-PCR primers

MdZDS1_F	GTTTCCACCTGAACCGGAGC	qRT-PCR primers
MdZDS1_R	ATTTTCGTCTGCACCCACCT	qRT-PCR primers
MdZDS2_F	GAGGCCTTTCATTGGCGGAA	qRT-PCR primers
MdZDS2_R	AGAGCCACTGCATTTCTTGC	qRT-PCR primers
MdCRTISO_F	ATGCCCTTTTCCCACTCACT	qRT-PCR primers
MdCRTISO_R	TTCCCTGATAGCCATGCTGC	qRT-PCR primers
MdLCY-β_F	CTACCTGGTCTGGTGCTGTT	qRT-PCR primers
MdLCY-β_R	GCATCGAGAACCACAGAAGC	qRT-PCR primers
MdCHY-β1_F	ATTATAACGCTGCGTGCGTC	qRT-PCR primers
MdCHY-β1_R	ACTCCATCCCCACAGCAGAT	qRT-PCR primers
MdCHY-β2-1_F	ATGGAGGGCCAAAAACAGGT	qRT-PCR primers
MdCHY-β2-1_R	ACATAGGCACATTCCCACCC	qRT-PCR primers
MdCHY-β2-2_F	TTCCGGCAATAGCCCTTCTC	qRT-PCR primers
MdCHY-β2-2_R	ACACCCTCGAACTTCTCCGA	qRT-PCR primers
MdZEP_F	GATTGGTGGGCAATTCAAGCCT	qRT-PCR primers
MdZEP_R	AGAAAACTGCCGCTGATGGG	qRT-PCR primers
MdVDE_F	CCCAGATCCTGCTGCTCTTG	qRT-PCR primers
MdVDE_R	TGTATAGAATGCCGGGCTGG	qRT-PCR primers
MdCCS1_F	GCTGCGTTGATCCTTCTCCT	qRT-PCR primers
MdCCS1_R	AACCCCATTGCAGAGACACC	qRT-PCR primers
MdCCD1_F	ACTTGCAAATGCTAAGAGCAAAAC	qRT-PCR primers
MdCCD1_R	ATGGCGGTGCATGTGAGTAG	qRT-PCR primers
MdCCD4_F	ATCTATGCGCTTTGCGAGGT	qRT-PCR primers
MdCCD4_R	TAGGTGAGGAATGGACGCAC	qRT-PCR primers
MdNCED1_F	CTCCCCTTCCTATCAACAACCTTC	qRT-PCR primers
MdNCED1_R	TGTTTTGGGAAGTGGGGTTTG	qRT-PCR primers
MdNCED5_F	TCTTCAACGAGTGCGACGAG	qRT-PCR primers
MdNCED5_R	TTTCCTCCCGAGTAAGTTCCG	qRT-PCR primers
MdAP2-34_F (AD-EcoR1)	CGGAATTCATGGCGAAAACCTCACAGC	Yeast one hybrid
MdAP2-34_-R (AD-Xhol)	CCGCTCGAGTTATGCATCAAGTTCACAGTG	Yeast one hybrid
MdAP2-34_F (ORF, 62-SK)	CGGAATTCATGGCGAAAACCTCACAGC	LUC assay
MdAP2-34_R (ORF, 62-SK)	CCGCTCGAGTTATGCATCAAGTTCACAGTG	LUC assay
ProMdPSY1_F	TCTCAAAGTTGTCACTCGGGTG	Promoter (Y1H)
ProMdPSY1_R	ATCAAAAAATATAATTAATCTGCTGGGT	Promoter (Y1H)
ProMdPSY2-1_F	GGTGCAGTGTCAGACTAAATCTCG	Promoter (Y1H, LUC)
ProMdPSY2-1_R	TGGGTTTTAGGTTTTGAGTGTGAG	Promoter (Y1H, LUC)
ProMdPSY2-2_F	AGAGGGAAGAGAATCCTACTC	Promoter (Y1H)
ProMdPSY2-2_R	GTTTGTTTCAACTTGAAAGCAC	Promoter (Y1H)
ProMdPDS_F	AATCATGACTCTGACACAACTATTC	Promoter (Y1H)
ProMdPDS_R	TTTATCAAACAGGTTGCGCGC	Promoter (Y1H)
ProMdZDS1_F	GGATTCAAGCTTCTATAAACC	Promoter (Y1H)
ProMdZDS1_R	CAGGATTAAAACCCTAACATTC	Promoter (Y1H)
ProMdZDS2_F	AGCAGTGCACTGAGGTCGTCG	Promoter (Y1H)
ProMdZDS2_R	GGATTCAAGCCTCTAATAACCCAC	Promoter (Y1H)
ProMdLCY-β_F	AACTTGTCATGGCCTTGGACAC	Promoter (Y1H)
ProMdLCY-β_R	GAAATTCCAACACTTGTCCCG	Promoter (Y1H)
ProMdCHY-β2-1_F	ACAAAACAAATTAAAGACCGTTGAC	Promoter (Y1H)
ProMdCHY-β2-1_R	GGAAGTATGCTTGTTGGTGAGGT	Promoter (Y1H)
ProMdZEP_F	ACACGAACCAAGTACTCAATTC	Promoter (Y1H)
ProMdZEP_R	GGAGATCCTGAAGCCATGCGG	Promoter (Y1H)
ProMdCCD1_F	ACGGTTTGCGCCTACGCGTTTG	Promoter (Y1H)
ProMdCCD1_R	GTTGTTGTCATATTTCGATTGG	Promoter (Y1H)
MdAP2-34-F (GST-BamHI)	CGGGATCCATGGCGAAAACCTCACAGC	EMSA
MdAP2-34-R (GST-XhoI)	CCGCTCGAGTTATGCATCAAGTTCACAGTG	EMSA
MdPSY2-1_F (Hot probe)	CTTTTCTTCTCTCAGTACATGCACCGACGTCGATA TTAACCTTGGAACCT	EMSA

MdPSY2-1_R (Hot probe)	AGGTTCCAAGGTTAATATCGACGTCGGTGCATGT ACTGAGAGAAGAAAAG	EMSA
MdPSY2-1_F (Mutant cold probe)	CTTTTCTTCTCTCAGTACATGCAAAAACGTCGAT ATTAACCTTGGAACCT	EMSA
MdPSY2-1_R (Mutant cold probe)	AGGTTCCAAGGTTAATATCGACGTTTTTGCATGT ACTGAGAGAAGAAAAG	EMSA
MdPSY2-1_S1_F	GCTGGTGAAAGAACAGAGCAC	ChIP-PCR
MdPSY2-1_S1_R	AAGGAGGAGCAGCAGAAATCC	ChIP-PCR
MdPSY2-1_S2_F	TTCCATGTTACCAGCTATGCG	ChIP-PCR
MdPSY2-1_S2_R	AGTTTTTGCGTAACGACTGCG	ChIP-PCR
MdPSY2-1_S3_F	CCGTGAAAATTGTCGTGTCG	ChIP-PCR
MdPSY2-1_S3_R	TGAGCACGAGATGTTCGGAG	ChIP-PCR

Supplemental Table S2. Pearson's correlation between AP2s transcript levels and total carotenoid content in apple flesh of 'Benin Shogun' and 'Yanfu 3' . The * are statistically significant at *P* < 0.05, and * are statistically significant at P < 0.001.**

	Gene number	Apple cultivar	Relative expression level			Gene significance	
			120 d	150 d	170 d	Pearson *r*	*P* value
AP2s transcription factors	MD02G1176000 (MdAP2-07)	Benin Shogun	5.2366	3.3581	3.2974	-0.2089	0.6913
		Yanfu 3	1.0000	7.0083	7.2324		
	MD02G1265300 (MdAP2-23, MdAP2-50)	Benin Shogun	1.0985	1.2511	1.0000	-0.6166	0.1923
		Yanfu 3	2.2095	1.6153	1.4675		
	MD03G1044200 (MdAP2-10)	Benin Shogun	1.0000	1.4085	1.7604	0.7280	0.1009
		Yanfu 3	1.0527	1.4690	1.5496		
	MD03G1107900 (MdAP2-41)	Benin Shogun	1.0906	2.3727	4.1204	0.7876	0.0629
		Yanfu 3	1.0000	2.4143	2.8677		
	MD04G1105200 (MdAP2-24)	Benin Shogun	1.0000	2.6210	3.4950	0.8173*	0.0470
		Yanfu 3	1.7526	2.3657	1.4943		
	MD07G1099500 (MdAP2-33)	Benin Shogun	1.1820	1.0225	1.1492	-0.0285	0.9573
		Yanfu 3	1.0019	1.0000	1.7552		
	MD08G1176000 (MdAP2-22)	Benin Shogun	1.0000	1.5086	1.9028	0.7376	0.0942
		Yanfu 3	1.2324	1.0445	1.8384		
	MD12G1075200 (MdAP2-44, MdAP2-45)	Benin Shogun	2.7686	1.0000	1.1265	-0.8698*	0.0243
		Yanfu 3	2.1129	1.9919	2.4407		
	MD12G1079800 (MdAP2-13, MdAP2-30)	Benin Shogun	1.3798	1.6052	1.0763	0.0105	0.9843
		Yanfu 3	1.0000	1.4947	1.6651		
	MD12G1125900 (MdAP2-11)	Benin Shogun	2.3134	2.1936	1.0000	-0.3610	0.4820
		Yanfu 3	2.7207	2.8784	6.6960		
	MD15G1286400 (MdAP2-17, MdAP2-26)	Benin Shogun	1.0141	2.2474	2.6900	0.8073	0.0521
		Yanfu 3	1.5813	1.0000	2.4268		
	MD17G1226700 (MdAP2-34)	Benin Shogun	0.9986	9.8061	11.6411	**0.9844 *** **	0.0004
		Yanfu 3	1.0000	2.9651	5.9588		
